# Enhanced Edar Signalling Has Pleiotropic Effects on Craniofacial and Cutaneous Glands

**DOI:** 10.1371/journal.pone.0007591

**Published:** 2009-10-26

**Authors:** Shie Hong Chang, Stephanie Jobling, Keith Brennan, Denis J. Headon

**Affiliations:** 1 Faculty of Life Sciences, University of Manchester, Manchester, United Kingdom; 2 The Roslin Institute and Royal (Dick) School of Veterinary Studies, University of Edinburgh, Edinburgh, Midlothian, United Kingdom; State University of New York College at Oneonta, United States of America

## Abstract

The skin carries a number of appendages, including hair follicles and a range of glands, which develop under the influence of EDAR signalling. A gain of function allele of *EDAR* is found at high frequency in human populations of East Asia, with genetic evidence suggesting recent positive selection at this locus. The derived *EDAR* allele, estimated to have reached fixation more than 10,000 years ago, causes thickening of hair fibres, but the full spectrum of phenotypic changes induced by this allele is unknown. We have examined the changes in glandular structure caused by elevation of Edar signalling in a transgenic mouse model. We find that sebaceous and Meibomian glands are enlarged and that salivary and mammary glands are more elaborately branched with increased Edar activity, while the morphology of eccrine sweat and tracheal submucosal glands appears to be unaffected. Similar changes to gland sizes and structures may occur in human populations carrying the derived East Asian *EDAR* allele. As this allele attained high frequency in an environment that was notably cold and dry, increased glandular secretions could represent a trait that was positively selected to achieve increased lubrication and reduced evaporation from exposed facial structures and upper airways.

## Introduction

The skin acts as a barrier to the immediate environment, relying in particular on its outermost layer of dead cells to protect the body from desiccation and infection. However, living tissues require some direct contact with the environment for exchange of nutrients, water and air. These external contacts are facilitated by a battery of glands that produce a diverse array of secretions and carry out very diverse functions. The mammary glands are used to nourish offspring, human eccrine sweat glands for thermoregulation, and the craniofacial glands of eyes, nose, mouth and upper airway act to lubricate and humidify the living tissues exposed to the exterior. In addition to maintaining tissue moisture, many glandular secretions also act as a barrier to infection [1].

The basic structure of a gland consists of an epithelial sheet, which contains the cells responsible for producing and secreting the glandular product, and a supporting connective tissue carrying blood vessels. Glands are described as being simple if the epithelium forms a single approximately spherical or cylindrical structure, such as the eccrine sweat glands. Glands that are required to secrete large volumes into a limited number of ducts or in a short space of time (e.g. saliva, milk, tears) are more complex, having a branched epithelium to increase the amount of secreting epithelial surface within a given volume of tissue [1].

Though cutaneous glands have diverse forms and roles in the adult, they all initiate development by budding off the surface covering of the embryo, called the ectoderm. This common developmental basis is underlain by a common genetic basis, with the result that single gene mutations can have pleiotropic effects on multiple glands, and also on the distribution and structure of teeth and hairs. Mutations affecting signalling through the cell surface receptor EDAR (OMIM#604095), either by mutation of *EDAR* itself or of the genes encoding its interaction partners EDA or EDARADD, cause hypohidrotic ectodermal dysplasia (also known as anhidrotic ectodermal dysplasia or Christ-Siemens-Touraine syndrome) [2]. Outwardly, hypohidrotic ectodermal dysplasia (HED) in humans is characterised by the presence of sparse hair, the absence of many teeth and the peg-like or conical shape of the teeth that do develop. In addition to these externally visible characteristics, HED also results in an absence of eccrine sweat glands and the reduction or absence of the many glands that secrete onto the surfaces of the craniofacial region (eyes, nasal passage, mouth and upper airway). These morphological changes result in increased risk of hyperthermia due to the inability to sweat, and in dryness, irritation and recurrent infection of the eyes, mouth, nose and upper airway due to a reduction of the secretions that normally lubricate and protect these tissues [3], [4]. These symptoms are managed in HED patients by the application of oral, nasal and ocular lubricants [5], [6], [7]. Mouse models of HED caused by mutations affecting Edar function display the same morphological features as those observed in the human condition [8], [9] and have been useful in identifying its developmental and genetic basis [10], [11].

In addition to null mutations that cause disease, less extreme modulation of EDAR pathway components appears to play a role in the striking variation of skin appendage forms across the vertebrates [12]. In humans, a non-synonymous SNP (rs3827760:T>C), which causes a p.Val370Ala substitution in the death domain of EDAR, is found at high frequency in East Asian and Native American, compared to European, other Asian and African, populations [13], [14], [15], [16] (Table S1). In addition to the extreme population differentiation at rs3827760, the unusual haplotype structure surrounding this SNP has been interpreted as evidence of recent positive selection at this locus in East Asia [17], [18], [19], [20]. However, demographic processes can leave traces that mimic selection signatures, questioning the ability of statistical methods based on population genetic data alone to identify loci that have undergone positive selection [21], [22]. Thus, following their initial identification in genomewide scans, putative positively selected loci should be examined for associated phenotypic effects and the altered trait(s) placed in the context of a biological hypothesis in which they could be, or have been, of benefit. In the case of *EDAR*, the derived allele encoding EDAR370A has been reported to display increased signal potency *in vitro* [13], [23], though Fujimoto et al. reported reduced signal intensity from this receptor variant [14]. Association studies have found that EDAR370A makes a major contribution to the increased hair fibre thickness observed in East Asian compared to other human hair forms [14], [24]. This thickened, coarse hair phenotype is replicated in mice genetically modified to undergo increased Edar signalling [23].

Thus rs3827760 is associated with a marked effect on EDAR protein function and, to date, one phenotypic trait. However, it is unclear what benefit could have been conferred by thickening of hair fibres to explain positive natural selection for this allele. Here we use a mouse model to assess the effects of increased Edar signalling on cutaneous glands and suggest that altered glandular function should be considered as a potential target for prehistoric selection on rs3827760 in East Asia.

## Results

We assessed the size and structure of glands in wild type (i.e. non-transgenic), *Edar^Tg951^* heterozygous transgenic and *Edar^Tg951/Tg951^* homozygous transgenic animals. In this transgenic line increased Edar signalling arises from a high copy number of the wild type *Edar* locus, causing elevated expression under control of endogenous regulatory elements. Heterozygous transgenic mice have a hair fibre thickness intermediate between that of wild type and homozygous transgenics [23], as observed in humans heterozygous at rs3827760 [24], demonstrating a dose-effect of Edar signalling on hair thickness.

**Figure 1:**
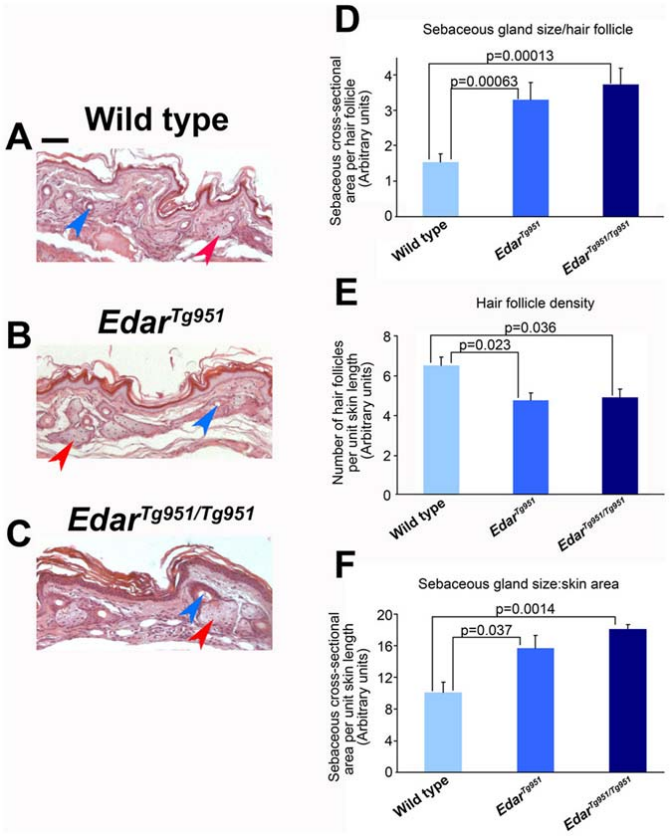
Increased sebaceous gland size in transgenic mice with elevated Edar signalling. (A–C) Haematoxylin & eosin stained sections of the dorsal region of the hindfeet. A subset of sebaceous glands is indicated by red arrowheads and hair canals by blue arrowheads. (D) Quantification of cross-sectional area of sebaceous glands normalised to hair follicle number. (E) Hair follicle density in wild type and transgenic skin. (F) Quantification of cross-sectional area of sebaceous glands normalised to skin area. Scale bar indicates 50 µm.

The sebaceous glands are associated with hair follicles and secrete sebum composed of a complex combination of lipids [25]. We examined the effects of increased Edar signalling on sebaceous gland size by measuring total gland area on sectioned skin stained with haematoxylin and eosin, a stain combination widely used to reveal tissue structure. This procedure does not stain the lipid component of the sebocytes, giving the glands a characteristic appearance (Figure 1A–C). Measurement of gland sizes on tissue sections showed that the sebaceous glands associated with each hair follicle are larger in transgenic than in wild type animals (Figure 1D). However, the large hair follicles of *Edar* transgenic animals are present at low hair density in the skin (Figure 1E), consistent with the reduced hair follicle density in human East Asian populations [26], [27], [28]. Due to this reduction in hair density in transgenic animals, we went on to determine the aggregate sebaceous gland size per unit area of skin, finding this to be greater in transgenic than in wild type animals (Figure 1F). Thus the enlargement of sebaceous glands upon increasing Edar signalling more than compensates for the accompanying reduction in hair follicle density, yielding a greater volume of sebaceous glands for a given area of skin.

**Figure 2:**
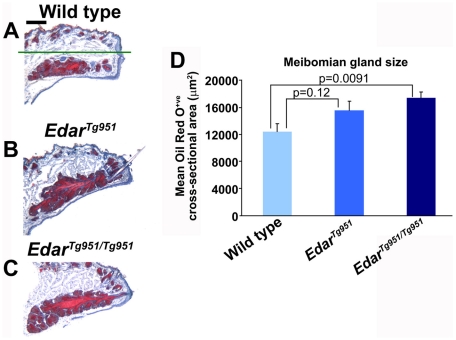
Increased Meibomian gland size in Edar transgenic animals. (A–C) Oil Red O stained eyelids of wild type, *Edar^Tg951^* and *Edar^Tg951/Tg951^* animals. Haematoxylin is used as a counterstain to reveal tissue structure. The sebaceous glands of the outer eyelid are above the green line in (A), while the Meibomian gland is below this line. (D) Quantification of Meibomian gland cross-sectional area on sampled slides for each genotype. The difference between wild type and *Edar^Tg951^* values did not achieve statistical significance in these analyses. Scale bar indicates 200 µm.

The Meibomian or tarsal glands are specialised enlarged sebaceous glands in the eyelid that secrete lipid onto the surface of the eye. This lipid coats the very thin layer of aqueous tear film, greatly reducing its rate of evaporation [29], [30]. These glands are absent in humans and corresponding mouse models of hypohidrotic ectodermal dysplasia [6], [31]. We stained the Meibomian glands on sections of eyelid using Oil Red O, a stain specific to lipids (Figure 2A–C) and measured gland profile areas from these sections. These analyses found that homozygous *Edar^Tg951/Tg951^* transgenic animals displayed glands that are significantly larger than those of the wild type controls (Figure 2D). Increased size of sebaceous and Meibomian glands has previously been observed upon overexpression of *Eda* from a heterologous promoter [32], [33], though these lines have not been reported to display the characteristic hair fibre thickening observed upon enhancement of *EDAR* function in mouse and human [14], [23].

**Figure 3:**
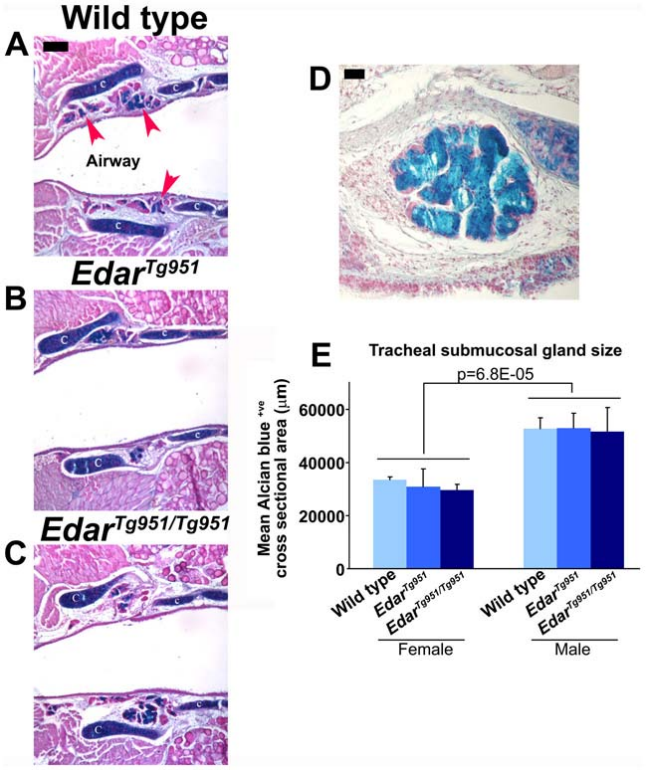
Sex differences, but not Edar-induced alterations, in tracheal submucosal gland size. (A–C) Sectioned tracheae stained with Alcian blue and counterstained with nuclear fast red. The cartilage of the trachea and the mucous of the submucosal glands stain blue. Submucosal glands are indicted by red arrowheads in (A), while the cartilages of the trachea are labelled ‘c’. (D) Magnified view of stained wild type tracheal submucosal gland. (E) Quantification of tracheal submucosal gland sizes. Scale bar A–C indicates 200 µm; scale bar D indicates 50 µm.

The tracheal submucosal glands secrete mucous onto the surface of the upper airway, serving to humidify inspired air and to protect tissue from desiccation and pathogens [34]. These glands are absent in humans [35] and mice [36] with abolished EDAR function. In order to determine the effect of elevating Edar signalling on the size of these glands, we sectioned mouse tracheae and stained the sections with Alcian blue. This stain recognises mucopolysaccharides and glycosaminoglycans, resulting in staining of mucous within glands and also the cartilage rings of the trachea (Figure 3A–D). Measurement of gland areas on sampled sections revealed a marked sexual dimorphism in their sizes, but no difference was detected between transgenic and wild type animals (Figure 3E).

**Figure 4:**
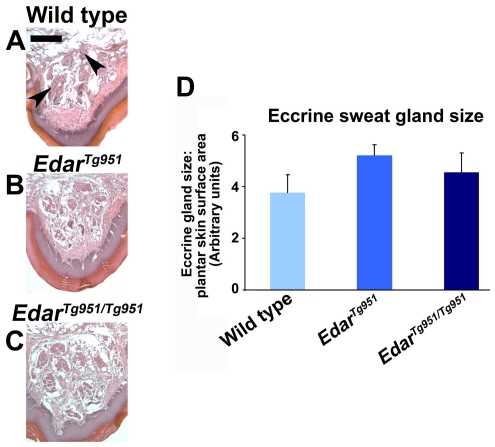
Lack of detectable changes to eccrine sweat gland morphology upon elevation of Edar signalling. (A–C) Haematoxylin and eosin stained ventral hindfoot. Eccrine glands in (A) are indicated by black arrowheads. (D) Quantification of eccrine sweat gland size per unit footpad skin length. Statistical significance was not attained between any two genotypes, nor did combining *Edar^Tg951^* and *Edar^Tg951/Tg951^* values yield a statistically significant difference when compared to wild type. Scale bar indicates 200 µm.

Eccrine glands produce an aqueous secretion, used by humans for thermoregulation by producing sweat across the entire skin. Other mammals do not use eccrine glands for thermoregulation, and carry these glands only in naked skin surfaces exposed to friction, such as the foot pads [37]. Despite the difference in the distribution of eccrine sweat glands in human compared to mouse skin, abolition of EDAR function in either species causes these glands to be entirely absent [8]. We examined the structure and size of the eccrine glands in the mouse plantar (hindfoot pad) skin by measuring gland areas on sectioned skin stained with haematoxylin and eosin. The eccrine glands are simple coiled tubes that appear as epithelial clusters in sectioned footpads (Figure 4A–C). Our analyses did not detect a statistically significant difference in the total size or distribution of eccrine glandular structures in animals with elevated Edar signalling (Figure 4D).

**Figure 5:**
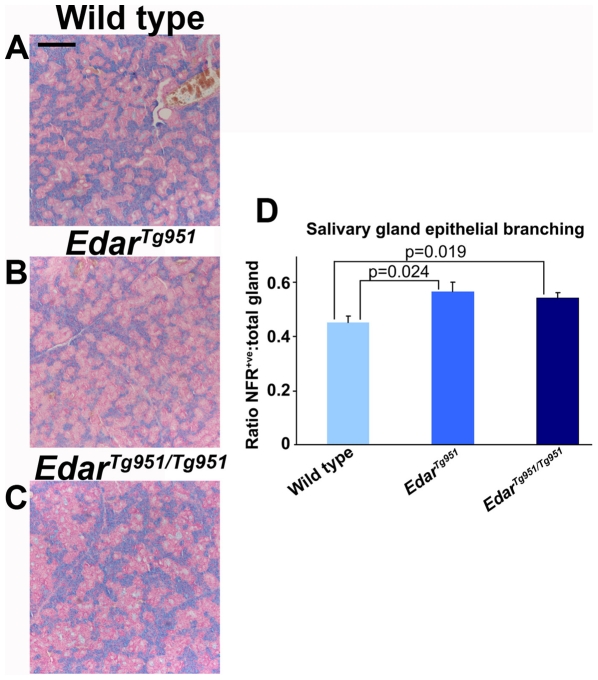
Enhanced Edar signalling produces increased epithelial branching of adult salivary glands. (A–C) Alcian blue and nuclear fast red stained salivary gland sections. (D) Quantification of the epithelial duct proportion of the salivary gland by determination of nuclear fast red stained (denoted NFR^+ve^) area to total gland area. Scale bar indicates 400 µm.

Saliva lubricates the mouth and throat, helps to form a bolus of chewed food for swallowing, initiates digestion of starch and lipids and aids in humidification of inspired air [38]. In the embryo, salivary glands are formed by epithelial ingrowth from the surface ectoderm followed by repeated epithelial branching, producing a large surface area of secretory tissue and a ductal structure that guides saliva to the oral cavity [39]. Salivary glands are present in HED mice, though their embryonic development is poor [40], and these glands are also present in human HED sufferers, though they produce less saliva than normal and the saliva produced has an altered chemical composition [41]. We stained sectioned submandibular salivary glands with Alcian blue, to stain mucous producing cells, and nuclear fast red to stain cell nuclei. This stain combination highlighted the epithelial ducts of the salivary gland (Figure 5A–C). We determined the frequency of epithelial ducts in the gland by measuring the ratio of nuclear fast red stained tissue to the total area of the gland on tissue sections. This analysis revealed a significant difference in glandular structure between adult *Edar* transgenic and wild type animals, with a greater degree of epithelial branching in the transgenic glands (Figure 5D).

**Figure 6:**
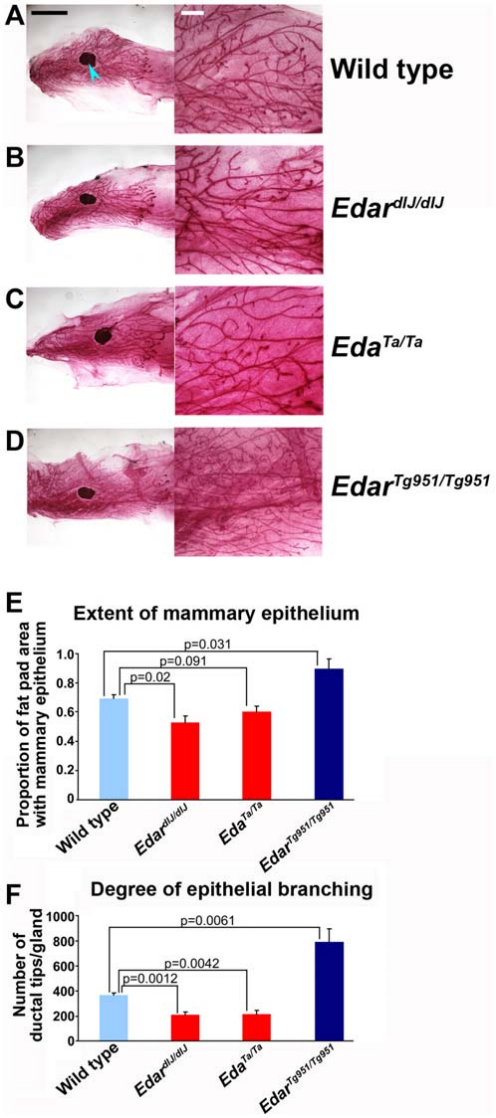
Mammary gland branching and epithelial growth extent is stimulated by Edar signalling. Whole mount stained mammary glands of 6 week old (A) wild type, (B) *Edar^dlJ/dlJ^* loss of function mutant, (C) *Eda^Ta/Ta^* loss of function mutant and (D) *Edar^Tg951/Tg951^* gain of function transgenic. The lymph node within the fat pad is indicated by a blue arrowhead in (A). (E) Quantification of the extent of mammary epithelial infiltration into the fat pad. (F) Determination of epithelial branching, expressed as the total number of ductal termini per mammary gland for each genotype. Comparison of mutant to transgenic mammary gland morphometric measures gave p-values <0.01. For A-D the black scale bar on the left panels indicates 5 mm and the white scale bar on the right panels indicates 1 mm.

The mammary glands are formed in a manner similar to that of the salivary glands by the repeated branching of an ingrowing epithelial cord [42]. Mammary gland function is poor in women with HED [4], but mouse models of HED have no obvious difficultly in producing sufficient milk to feed a litter of pups (DJH, personal observation). We analysed the structure of the mammary glands of 6 week old virgin mice by whole mount staining to detect the epithelial cords (Figure 6A–D). This age and reproductive state was chosen as the functional, lactating gland is so densely packed with epithelium that quantitative analyses become difficult. We first examined the mammary structure in mutant animals with loss of Edar signalling, detecting a reduced mammary tree in both extent and degree of branching in the *Edar^dlJ^* [10] and *Eda^Ta^* [43] mutant lines (Figure 5B,C,E,F). Thus the consequences of abolished Edar function on mammary morphology are readily detected in these mutant mice, despite the absence of an apparent functional defect in lactation. Homozygous transgenic animals with elevated Edar signalling displayed a clear elaboration of mammary structure relative to wild type, with both greater extent of epithelial penetration into the fat pad and a greater density of epithelial branching (Figure 5D–F).

## Discussion

These results show that most of the structures that are reduced or fail to develop in the absence of Edar signalling are enlarged or elaborated with increased signalling. However, this is not an absolute rule as we did not detect morphological changes in eccrine sweat or tracheal submucosal glands, both of which are absent when Edar function is abolished. The functional effects of the observed increased size of simple glands (Meibomian and sebaceous) and the increased epithelial component of branched glands (salivary and mammary) in the adult are likely to include an increase in the rate, and possibly altered composition, of glandular secretions.

The congruence of *EDAR* loss of function phenotypes in human and mouse [10], [11], and the very similar effects on hair morphology caused by elevated EDAR signalling in both species [14], [23], indicate that the mouse represents a good model for studying EDAR function in human. Thus morphological changes of a direction similar to those reported here in the mouse are likely to occur in the glands of humans expressing EDAR370A. This suggests that rs3827760 may contribute to phenotypic variation in glandular structure and function among modern human populations. For example, among women living in the United States mammographic breast density is reported to be greater in those with Chinese and Japanese ancestry than in those of European and African descent [44], consistent with our observation of increased epithelial density in Edar transgenic mice.

The derived *EDAR* allele is estimated to have reached fixation greater than 10,000 years ago [13], probably in Northeast Asia based on the high present day frequency of the allele in this region and in Native Americans, indicating its prevalence in the population that travelled across Beringia [19]. If the derived *EDAR* allele rose to high frequency as a result of positive natural selection then it should produce a trait that was beneficial in this location and at this time. In eastern and northern Asia the climate between 25,000 and 10,000 years ago was for most of this period significantly colder and drier than at present [45], [46], [47] and it has long been hypothesised that modern East Asian populations are derived from ancestors with morphological and physiological adaptations to cold, dry conditions [48], [49], [50]. Altered hair form or mammary function can not readily be linked to functions specifically required by humans for adaptation to a dry, cold environment; conditions that increase evaporation from exposed surfaces, leading to drying of the eyes, nose, mouth, throat and skin, and to cooling of the skin [51]. Based on their known physiological functions, the increased activity of craniofacial glands could be beneficial in such conditions.

The Meibomian glands secrete lipid onto the aqueous tear film of the eye to slow its rate of evaporation [29], the rate of tear evaporation being increased in conditions of low humidity [52]. Reduced Meibomian gland function causes dry-eye symptoms of discomfort, inflammation and tissue damage [29]. Thus increasing Meibomian secretions could help protect the tear film from evaporation in a low humidity environment by thickening its protective lipid layer. Alteration of eyelid shape to the typical East Asian form reduces the area of the eye exposed to the air, and this is observed to reduce tear film evaporation [53]. Thus altered eyelid aperture and increased Meibomian gland secretions could represent independent adaptations to prevent water loss from the eyes. Conditions of low humidity lead to drying of the mouth and throat [54] and increased salivation potential from more highly branched glands could aid in maintaining humidification and protection from infection in dry conditions. Enlargement of sebaceous glands may also contribute to adaptation to dry, cold conditions. These glands secrete sebum that coats hair fibres and the skin surface. In human skin the sebaceous glands are found mostly on the scalp and face [55]. Studies on mice have shown that sebum has a profound effect on the skin; the absence of sebaceous gland function leading to inflammation and dryness of the outermost cornified layer of skin [56] and to dramatic loss of water and heat through the skin, rendering sebum-deficient animals highly susceptible to hypothermia in cold conditions [57], [58]. The major skin humectant derived from the sebaceous glands is glycerol, which represents an important endogenous moisturiser in dry and cold conditions [59]. Thus sebum acts to aid retention of moisture within the skin, conserve body water and reduce heat loss in conditions of low humidity and temperature. Due to the distribution of the glands these effects would be greatest on the face and scalp, which are likely to be the most exposed areas of skin for clothed humans. The action of sebaceous glands also appears to be important in human development and the abrupt transition to a dry air environment at birth. Foetal sebaceous glands are activated in utero to produce a sebum covering, the vernix caseosa, which has important water barrier and anti-infective properties [60]. Together, the modification of these craniofacial glands is likely to provide an ability to increase aqueous secretions onto mucosae and to protect tissue moisture from evaporation by use of lipid films.

Based on (i) the enlargement and elaboration of craniofacial and cutaneous glands caused by elevated Edar signalling in the mouse, (ii) the climate in Pleistocene East Asia as the derived *EDAR* allele went to fixation and (iii) the clinical features of abolished EDAR activity in ectodermal dysplasia, it appears that selection for increased lubrication and humidification of exposed surfaces represents a plausible adaptive trait in East Asia during the ice age. This scenario would imply that alteration of hair form, mammary structure and any possible effect on tooth morphology, are ‘off-target’ consequences of pleiotropic *EDAR* action. Population growth and migrations subsequent to this ancient episode of selection would explain the present day distribution of this allele across a wide variety of climactic conditions [19], notably in the Americas.

We note speculative nature of the hypothesis we introduce, which requires (i) that at least some of the effects we observe on mouse glands occur in humans carrying the derived *EDAR* allele and (ii) that these effects alter gland function to provide some physiological benefit in cold, dry conditions. Here our aim is to provide data from mouse to inform genetic association studies in modern human populations. Any association of rs3827760 with altered gland function should be considered in light of other alleles at high frequency in East Asia, such as an apparent gain of function allele of *FGFR2* [61], a key stimulator of sebaceous gland activity [62], a null allele of *ABCC11*, which reduces secretions from apocrine type (mammary, ceruminous and axillary) glands [63], [64], and altered endocrine parameters [65], [66], [67], contributing to the unique glandular phenotype that has arisen in this region.

## Materials and Methods

### Ethics statement

All animal work conformed to guidelines for animal husbandry at the University of Manchester and was carried out under UK Home Office licence.

### Animals

Wild type, transgenic and mutant animals were on an inbred FVB/N genetic background. *Edar^Tg951^* transgenic mice carry approximately 19 copies of a 200 kb yeast artificial chromosome (YAC) containing the entire mouse *Edar* gene, while homozygous *Edar^Tg951/Tg951^* animals carry 36 copies [23]. Tissues from eight week old animals were used for all analyses apart from those of the mammary gland, for which six week old animals were used. Morphometric values for male and female gland parameters are combined, except in the case of the tracheal submucosal glands, where a sexual dimorphism in gland size was detected.

### Tissue processing and sectioning

Tissues were fixed and stored in 4% formal saline at 4°C. Right hind feet, used for determination of eccrine and sebaceous gland sizes, were decalcified using formic acid. Dehydrated, paraffin-embedded tissues were cut at 20 µm (tracheae), 10 µm (salivary glands) or 8 µm (eccrine and sebaceous glands). For Oil Red O staining of Meibomian glands, right eyelids were embedded in OCT tissue-freezing medium (Thermo Scientific) and 20 µm sections cut at −20°C in the sagittal plane. Slides were air-dried at room temperature before staining or stored at −80°C until needed.

### Histochemical staining

Sections of the tracheal and submandibular glands were deparaffinised in histoclear, rehydrated in graded alcohols, stained with Alcian blue solution (1% Alcian blue, 3% acetic acid) for 5 minutes, washed in water, and counterstained with nuclear fast red (0.1% nuclear fast red, 5% aluminium sulphate) for 5 minutes, followed by washing with water. Sections were then dehydrated, cleared in xylene, and mounted in Pertex Mounting Medium.

For Oil Red O staining of Meibomian glands, frozen sections of the eyelid were washed with running water and rinsed with 60% isopropanol. Sections were stained with 0.5% Oil Red O (Sigma) in isopropanol at room temperature for 15 minutes, followed by a 60% isopropanol rinse. Sections were counterstained with haematoxylin, washed with distilled water and mounted in aqueous mountant.

### Morphometric measurements from sectioned tissues

Measurements of gland sizes and morphologies were performed on digital images using Image-Pro 6.2 software (MediaCybernetics), with the exception of mammary gland parameters, which were measured using Image J 1.40 g software. Measurements were done on four to six animals per genotype.

To quantify tracheal submucosal gland sizes, serial sections were collected through the entire trachea, which was sectioned from dorsal to ventral aspects. All sections were mounted and stained, with 4 sections per slide, and gland area was measured from one section on each slide collected.

To determine Meibomian gland size, serial frozen sections of right eye eyelids (upper and lower together) were taken, with 10–30 sections collected onto each slide. For every 10^th^ section Meibomian gland area was quantified by measuring the area stained with Oil Red O, excluding the region of the eyelid carrying hair follicle-associated sebaceous glands.

To determine submandibular salivary gland structure, 1–5 sections were collected onto each slide and one section was randomly selected for analysis. At least eight slides from each animal were used for these determinations. Image-Pro 6.2 software was used to calculate the area stained with nuclear fast red, and the area stained with Alcian blue. The ratio of duct to total area, i.e. [pink/( pink+blue)] was used to represent the proportion of duct within the gland.

To determine eccrine gland sizes, tissue sections from three locations at the footpads and proximal digits of the hindfoot were collected and gland area (including secretory portion and ducts) and plantar epidermal length were measured at each location. Sebaceous gland sizes were determined by measuring gland area on the dorsal aspect of the hindfoot sections.

### Mammary gland whole mount staining and morphometrics

The rostral inguinal (4^th^) mammary glands were flattened onto microscopic slides and fixed overnight in Carnoy's solution (60% ethanol, 30% chloroform and 10% glacial acetic acid). Glands were rehydrated and stained overnight with carmine alum (2% carmine dye, 5% aluminium potassium sulphate). They were subsequently dehydrated, incubated in xylene for 1 hour, and left in methyl salicylate (Sigma) for storage. To quantify mammary gland epithelial morphology, epithelial infiltration was measured by calculating the total area of fat pad penetrated by ductal epithelium, and ductal branching was calculated by counting the total number of ductal termini per gland.

### Statistical analyses

Pairwise t-tests were performed for results from each genotype and gland type. Unless indicated in the figure legend, all p-values below 0.2 are displayed in the figures. More detailed statistical information is presented in Table S2.

## Supporting Information

Table S1 Allele frequencies of rs3827760 in 7 populations. Data are taken from HapMap Phase II+III (www.hapmap.org). For a more detailed map of regional rs3827760 allele frequencies see Sabeti et al. [19].
(0.03 MB DOC)

Table S2 Statistical information related to data sets presented in the figures.
(0.07 MB DOC)
