# Supplementary material for: Enhanced Edar Signalling Has Pleiotropic Effects on Craniofacial and Cutaneous Glands
Source: PLoS One. 2009 Oct 26;4(10):e7591. doi: 10.1371/journal.pone.0007591 (PMC2762540; doi:10.1371/journal.pone.0007591)
Supplement: Table S1 — Allele frequencies of rs3827760 in 7 populations. Data are taken from HapMap Phase II+III (www.hapmap.org). For a more detailed map of regional rs3827760 allele frequencies see Sabeti et al. [19]. (0.03 MB DOC) [file pone.0007591.s001.doc]

| **Population description** | **Frequency of ancestral rs3827760 allele (encoding EDAR370V)** | **Frequency of derived rs3827760 allele (encoding EDAR370A)** |
| --- | --- | --- |
| Utah residents with Northern and Western European Ancestry | 1.0 | 0 |
| Han Chinese in Beijing | 0.06 | 0.94 |
| Chinese in Denver, Colorado | 0.08 | 0.92 |
| Gujarati Indians in Houston, Texas | 0.98 | 0.02 |
| Japanese in Tokyo | 0.2 | 0.8 |
| Mexican ancestry in Los Angeles, California | 0.59 | 0.41 |
| Yoruba in Ibadan, Nigeria | 1.0 | 0 |

**Table S1. Allele frequencies of rs3827760 in 7 populations.** Data are taken from HapMap Phase II+III ([www.hapmap.org](http://www.hapmap.org/)). For a more detailed map of regional rs3827760 allele frequencies see Sabeti et al. [19].
