# Supplementary material for: Enhanced Edar Signalling Has Pleiotropic Effects on Craniofacial and Cutaneous Glands
Source: PLoS One. 2009 Oct 26;4(10):e7591. doi: 10.1371/journal.pone.0007591 (PMC2762540; doi:10.1371/journal.pone.0007591)
Supplement: Table S2 — Statistical information related to data sets presented in the figures. (0.07 MB DOC) [file pone.0007591.s002.doc]

| **Gland measurement** | **Genotype** | **n** | **Mean** | **Std dev** | **p-value vs. Tg/+** | **p-value vs. Tg/Tg** | **p-value vs. All Tg** | **p-value vs. Edar^dlJ^** | **p-value vs. Eda^Ta^** |
| --- | --- | --- | --- | --- | --- | --- | --- | --- | --- |
| **Sebaceous size*** | WT | 4 | 1532.37 | 232.0254 | 0.000633 | 0.000128 | 2.11E-05 |  |  |
|  | Tg/+ | 4 | 3303.059 | 493.3393 |  | 0.23994 |  |  |  |
|  | Tg/Tg | 4 | 3739.303 | 451.7889 |  |  |  |  |  |
| **Meibomian size (μm)** | WT | 5 | 124496.9 | 26747.75 | 0.122919 | 0.009126 | 0.014061 |  |  |
|  | Tg/+ | 5 | 155934.3 | 30762.81 |  | 0.278733 |  |  |  |
|  | Tg/Tg | 5 | 174762.5 | 19141.89 |  |  |  |  |  |
| **Submucosal size (μm)** | WT | 6 (3♀, 3♂) | 43252.3 | 11538.32 | 0.882965 | 0.76123 | 0.795227 |  |  |
|  | Tg/+ | 6 (3♀, 3♂) | 42059.7 | 15525.86 |  | 0.888037 |  |  |  |
|  | Tg/Tg | 6 (3♀, 3♂) | 40750.68 | 15870.34 |  |  |  |  |  |
| **Eccrine size*** | WT | 4 | 3.743993 | 1.406534 | 0.256852 | 0.474267 | 0.253007 |  |  |
|  | Tg/+ | 4 | 5.178507 | 0.817086 |  | 0.788314 |  |  |  |
|  | Tg/Tg | 4 | 4.5249 | 1.486372 |  |  |  |  |  |
| **Salivary branching** | WT | 5 | 0.453841 | 0.052726 | 0.024064 | 0.019132 | 0.006626 |  |  |
|  | Tg/+ | 5 | 0.568725 | 0.076039 |  | 0.53229 |  |  |  |
|  | Tg/Tg | 5 | 0.543173 | 0.043385 |  |  |  |  |  |
| **Mammary infiltration** | WT | 4 | 0.6955 | 0.051553 |  | 0.030721 |  | 0.02039669 | 0.0914995 |
|  | Tg/Tg | 4 | 0.89725 | 0.133974 |  |  |  | 0.00884183 | 0.0087691 |
|  | Edar^dlJ^ | 3 | 0.530333 | 0.080314 |  |  |  |  | 0.257808 |
|  | Eda^Ta^ | 4 | 0.605 | 0.073982 |  |  |  |  |  |
| **Mammary branching** | WT | 4 | 366.25 | 31.87868 |  | 0.00612 |  | 0.00123579 | 0.0042005 |
|  | Tg/Tg | 4 | 792.5 | 203.7408 |  |  |  | 0.00481311 | 0.0015727 |
|  | Edar^dlJ^ | 3 | 207.3333 | 31.50132 |  |  |  |  | 0.9148309 |
|  | Eda^Ta^ | 4 | 211.75 | 61.19572 |  |  |  |  |  |

* indicates arbitrary units of measurement

‘Tg/+' denotes *Edar^Tg951^* heterozygote

‘Tg/Tg' denotes *Edar^Tg951/Tg951^* homozygote

‘All Tg' denotes combined values of *Edar^Tg951^* and *Edar^Tg951/Tg951^*

**Table S2.** **Statistical information related to data sets presented in the figures.**
